# Assessing the Effects of Acute Amyloid β Oligomer Exposure in the Rat

**DOI:** 10.3390/ijms17091390

**Published:** 2016-08-24

**Authors:** Ryan S. Wong, David F. Cechetto, Shawn N. Whitehead

**Affiliations:** Vulnerable Brain Laboratory, Department of Anatomy and Cell Biology, Schulich School of Medicine and Dentistry, University of Western Ontario, 1151 Richmond St, London, ON N6A 5C1, Canada; ryanswong@gmail.com (R.S.W.); cechetto@uwo.ca (D.F.C.)

**Keywords:** Alzheimer’s disease, amyloid β oligomers, behavioural deficits, microglial activation, cholinergic neuron depletion

## Abstract

Alzheimer’s disease (AD) is the most common form of dementia, yet there are no therapeutic treatments that can either cure or delay its onset. Currently, the pathogenesis of AD is still uncertain, especially with respect to how the disease develops from a normal healthy brain. Amyloid β oligomers (AβO) are highly neurotoxic proteins and are considered potential initiators to the pathogenesis of AD. Rat brains were exposed to AβO via bilateral intracerebroventricular injections. Rats were then euthanized at either 1, 3, 7 or 21-days post surgery. Rat behavioural testing was performed using the Morris water maze and open field tests. Post-mortem brain tissue was immunolabelled for Aβ, microglia, and cholinergic neurons. Rats exposed to AβO showed deficits in spatial learning and anxiety-like behaviour. Acute positive staining for Aβ was only observed in the corpus callosum surrounding the lateral ventricles. AβO exposed rat brains also showed a delayed increase in activated microglia within the corpus callosum and a decreased number of cholinergic neurons within the basal forebrain. Acute exposure to AβO resulted in mild learning and memory impairments with co-concomitant white matter pathology within the corpus callosum and cholinergic cell loss within the basal forebrain. Results suggest that acute exposure to AβO in the rat may be a useful tool in assessing the early phases for the pathogenesis of AD.

## 1. Introduction

Alzheimer’s disease (AD) is one of the most common forms of dementia. Since the discovery of AD, our understanding of disease initiation and progression have revolved around amyloid β (Aβ) peptides [1,2,3,4]. Aβ peptides are derived from sequential cleavage of the amyloid precursor protein (APP) and can exist in a several forms, such as monomers, oligomers, protofibrils and fibrils [5,6,7,8,9,10,11]. Although much is known about the disease, the earliest triggers and pathogenesis of AD is still not fully understood.

The amyloid cascade hypothesis is still one of the leading theories on AD initiation and progression. [12]. This hypothesis posits that Aβ peptides and insoluble plaques initiate a cascade of pathological events leading to the aberrant phosphorylation of tau, neuronal loss and eventual dementia [13,14]. Indeed, Aβ plaques and neurofibrillary tau tangles are still considered the gold standard in terms of post-mortem disease confirmation [15]. Although evidence in the literature has supported the amyloid cascade hypothesis, it does not fully explain the progression of the disease.

More recent evidence has shown that amyloid plaques may not be the primary initiator of the pathogenesis of AD, largely based on the poor correlation between amyloid plaques and the severity cognitive impairment [16,17,18,19]. Furthermore, a study using post-mortem human brain analysis revealed the presence of amyloid plaques in six of the nine non-demented elderly patients [20]. In an animal study, transgenic mice containing the mutation for the human APP gene (*Tg2576*), did not show cognitive deficits until five months after extracellular deposition of amyloid plaques [21]. Together, these evidence suggests that amyloid plaques, although play a role in the pathogenesis of most AD cases, may not be entirely responsible and furthermore may not play a critical role in the pathogenesis of the prodromal phase of Alzheimer’s disease.

Increasing evidence supports the role of the oligomeric form of the Aβ peptide (AβO) as being the critical initiator of toxicity in the pathogenesis of AD. AβO has been shown to be more neurotoxic than the Aβ fibrils that form plaques. [22]. Unlike amyloid plaques, AβO in humans have been shown to correlate well with the severity of the disease, such as synaptic changes and neurodegeneration [18,23]. In a clinical study, AβO in human AD cortical brain extracts were approximately 12-fold higher than their age-matched control groups [16]. In another study, human AD cortical brain extracted AβO injected into rat brains impaired synaptic plasticity and learning behaviours as shown by the passive avoidance conditioning test [24]. These clinical data all suggest that AβO play a critical role in the pathogenesis of AD.

The neurotoxic effects of AβO have been established in many in vitro studies. When incubating AβO with hippocampal cells, AβO have been shown to co-localize with post-synaptic dendrites in vitro and disrupt long term potentiation (LTP) [25,26,27,28]. Other studies have suggested that AβO can activate microglial cells by binding to receptors and increase inflammatory responses [29,30,31]. In vivo studies have also supported the role of AβO in the pathogenesis of AD. Studies using transgenic AD animal models, such as the Tg2576 mice model, have detected AβO in the brain prior to plaque deposition, and its presence correlated with cognitive deficits in spatial learning and memory, as observed with the Morris water maze task [21,32,33]. Other studies have shown that injecting AβO into the rat brain can inhibit long term potentiation, which can then be abrogated by treating the rats with AβO-specific antibodies [34,35]. These studies have provided some insight into the effects of AβO in a physiological environment. However, our understanding of AβO in vivo is incomplete and requires further investigation.

To investigate the effects of AβO in vivo, we injected synthetic AβO in the lateral ventricles of the rat brain. Although synthetic AβO may be less potent than the naturally-secreted from cells or human-derived AβO, studies have shown similar neurotoxic effects [36,37]. Furthermore, synthetic AβO are accessible and can be reproduced in the lab following established, reproducible protocols. We assessed changes in spatial learning and memory using the Morris water maze task and exploratory and anxiety-like behaviours using the open field task. We assessed the pathological outcome by immunolabelling for Aβ, Ox-6 and IBA-1 for microglia and choline acetyl transferase (ChAT) for basal forebrain cholinergic neurons. To our knowledge this is the first in vivo study to perform a single injection AβO into the lateral ventricles and assess pathological and behavioural outcomes. Our study demonstrates, for the first time, that AβO resulted in a transient behavioural deficits, transient Aβ deposition and sustained microglia activation.

## 2. Results

### 2.1. Transient Deficits in Spatial Memory and Mild Anxiety-Like Behaviour in AβO-Injected Rats

**Figure 1:**
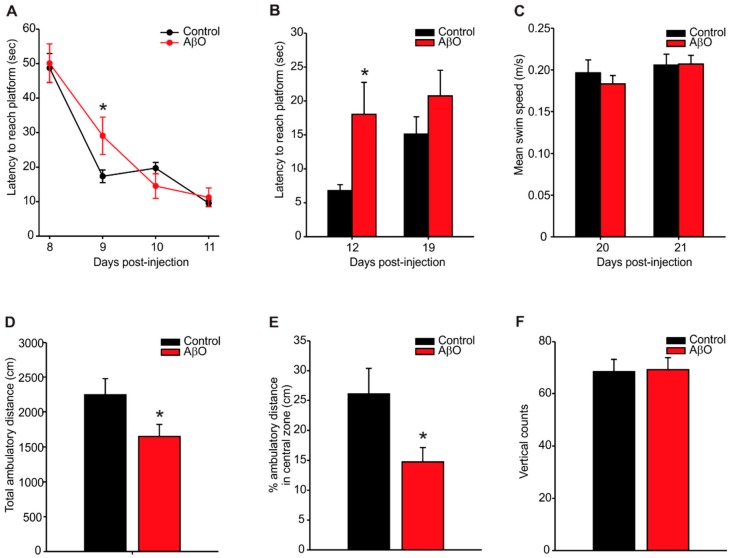
Behavioural assessment using the Morris water maze (**A**–**C**) and open field tasks (**D**–**F**). (**A**) Latency to reach platform was measured on post-surgical days 8–11 to assess initial learning of the task. AβO-injected rats took significantly longer to reach the platform on day 9 compared to control rats; (**B**) probe trials were performed on days 12 and 19 to assess memory. AβO-injected rats took significantly longer to reach the platform on day 12 compared to controls; (**C**) mean swim time indicated no differences in swim speed between AβO-injected and control rats; (**D**) AβO-injected rats showed significantly less ambulatory time compared to controls in the open field task; (**E**) AβO-injected rats spent significantly less time in the central zone compared to control rats; (**F**) no differences in motor ability detected using vertical counts between AβO-injected and control rats. Data presented as group means ± SEM. * indicates statistical significance between AβO-injected and control rats using 2-way ANOVA followed by Tukey’s post hoc analysis (**A**,**B**) and a 2-tailed students *t*-test (**D**,**F**), *p* < 0.05, *n* = 8 for each experimental group.

The Morris water maze task was used to determine whether exposure to AβO in rats would result in deficits in spatial learning and memory (Figure 1A–C). During the spatial learning phase, both the AβO-injected and control (PBS-injected) rats were able to successfully learn the location of the hidden platform, however the AβO-injected rats performed poorer, as indicated by a longer latency to reach the platform (control—17.35 ± 1.86 s, AβO—29.06 ± 5.40 s) on day 9 compared to control rats (Figure 1A). Probe trials at post-surgery day 12 and 19 was performed to assess any effects on short- or long-term spatial memory respectively (Figure 1B). AβO-injected rats took significantly longer to reach the platform on probe trial day 12 than the control rats (control—6.81 ± 0.85 s, AβO—18.01 ± 4.71 s). No differences in latency to reach the platform were observed by probe trial day 19. To insure that the differences in task performance wasn’t caused by motor, or other physiological deficits unrelated to AβO exposure, cued learning trials were performed on days 20 and 21 post-surgery (Figure 1C). No differences in swim speed were detected between AβO- and PBS-injected rats.

On post-surgery day 21, rats were placed in an open field apparatus for twenty minutes to test for exploratory and anxiety-like behaviours (Figure 1D–F). AβO-injected rats demonstrated significantly less ambulatory travel distance compared to controls (Figure 1D, control—2245.53 ± 230.49 cm, AβO—1649.44 ± 172.99 cm) and specifically, significantly less ambulatory distance traveled in the central zone compared to controls (Figure 1E, control—26.06% ± 4.32%, AβO—14.73% ± 2.37%), indicating an anxiety-like thigmotactic behaviour.

### 2.2. Transient Deposition of Aβ Following AβO Injections

**Figure 2:**
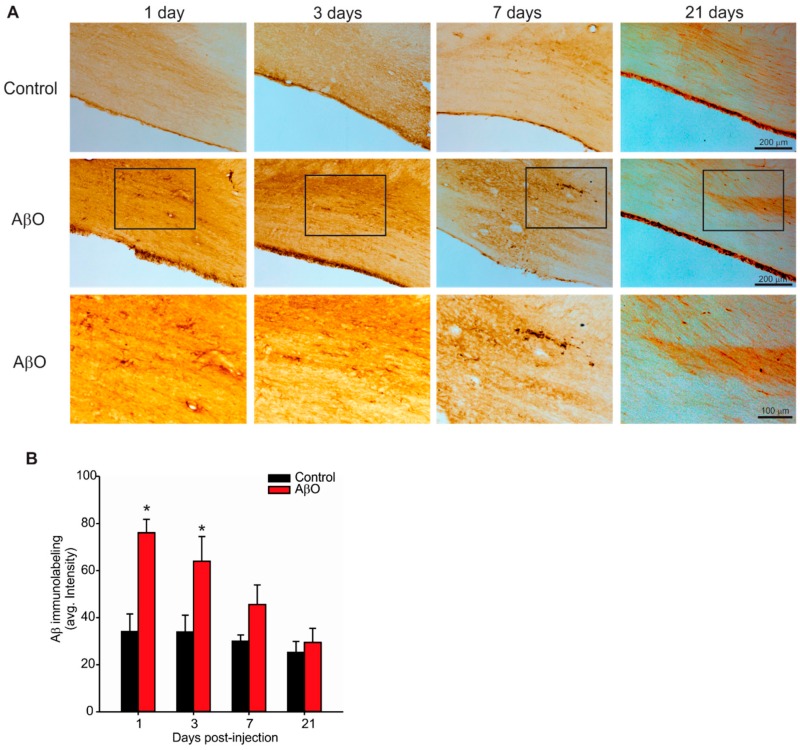
Immunolabelling for Aβ deposition within the corpus callosum and cingulate gyrus. Paraformaldehyde perfused rat brains were sectioned at 30 µm and stained with the Aβ4G8 antibody with an epitope against the 17–24 amino acid sequence of the Aβ peptide. (**A**) Photomicrographs of the corpus callosum and cingulate gyrus in coronal rat brain sections from AβO-injected and PBS-injected (control) rats 1, 3, 7 and 21 days post-injection. Bottom panels are higher magnification images of the panels immediately above (indicated by the box). Scale bars are 200 or 100 µm as indicated; (**B**) quantification using optical densitometry from three adjacent tissue sections per animal. AβO-injected rats had significantly more Aβ labelling in the corpus callosum and cingulate gyrus compared to controls at days 1 and 3 post-injection. Data presented as group means ± SEM. * indicates statistical significance between AβO-injected and control rats using 2-way ANOVA followed by Tukey’s post hoc analysis, *p* < 0.05, *n* = 5 for each experimental group.

We next investigated whether or not the AβO injections would result in Aβ deposition in the brain as well as to identify where the deposition may occur, and for how long it might in the brain. To identify Aβ deposition, immunohistochemical stains were done with Aβ4G8 antibody, which recognizes the 17–24 amino acid sequence of the Aβ protein (Figure 2). There was only one region within the brain that demonstrated positive Aβ4G8 labelling—the corpus callosum and cingulate gyrus adjacent to the injection sites, but remote from the injection sites themselves. In this region a transient increase in Aβ4G8 labelling was observed in AβO-injected rats compared to controls. Quantification of Aβ4G8 labelling revealed a significant increase in signal both at 1 and 3 days post-injection compared to controls (Figure 1B). By 7 days post-injection there were no differences in Aβ4G8 labelling between AβO-injected rats and controls (Figure 1B) indicating that any Aβ deposition caused by the AβO-injection was likely cleared from the brain parenchyma.

### 2.3. Cholinergic Neuron Depletion within Basal Forebrain Following AβO Injections

**Figure 3:**
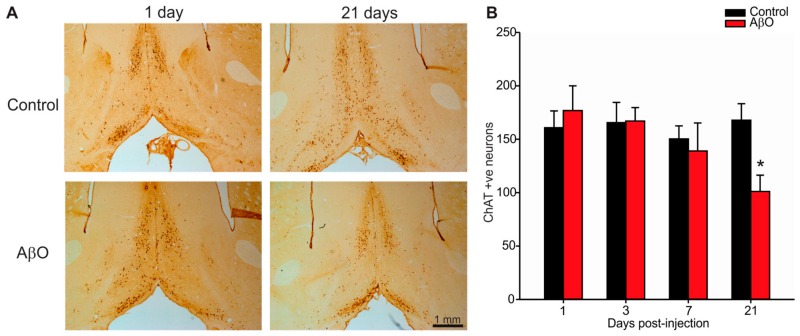
Immunolabelling for cholinergic neurons within the basal forebrain. Paraformaldehyde perfused rat brains were sectioned at 30 µm and stained with the ChAT antibody that specifically labels cholinergic neurons within the basal forebrain. (**A**) Photomicrographs of the basal forebrain in coronal rat brain sections from AβO-injected and PBS-injected (control) rats 1, and 21 days post-injection. Scale bar is 1 mm; (**B**) Quantification of cholinergic neuron cell counts from three adjacent tissue sections per animal. AβO-injected rats had significantly more ChAT labelling in the basal forebrain compared to controls 21 days post-injection. Data presented as group means ± SEM. * indicates statistical significance between AβO-injected and control rats using 2-way ANOVA followed by Tukey’s post hoc analysis, *p* < 0.05, *n* = 5 for each experimental group.

Pathological changes in AD patients include cholinergic neuron loss within the basal forebrain [38]. Specifically, cholinergic neuron loss occurs within the medial septal nucleus (MS) and vertical and horizontal diagonal bands of Broca of the basal forebrain. In this study cholinergic neurons were labelled with choline acetyltransferase (ChAT, Figure 3). By 21 days post-injection AβO-injected rats had significantly less ChAT positive neurons within the basal forebrain compared to controls (Figure 3B, control—26.06 ± 4.31 cells, AβO—14.73 ± 2.38 cells).

### 2.4. Microglia Activation in Response to AβO Injections

**Figure 4:**
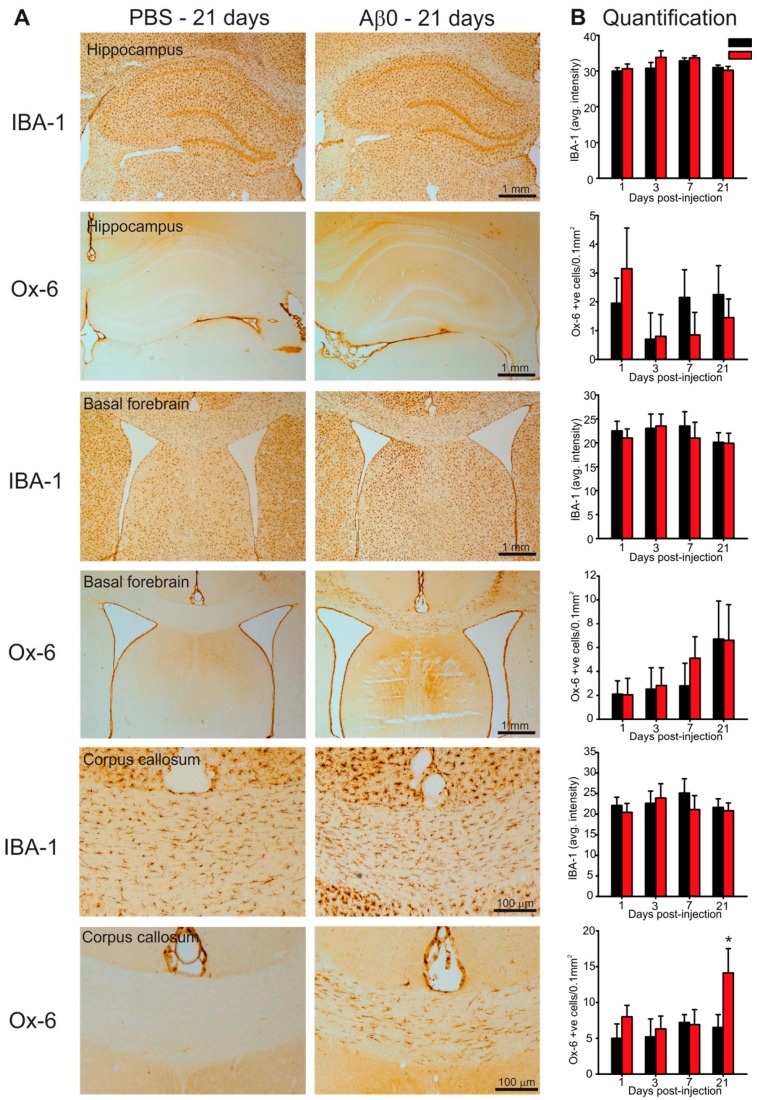
Microglia assessment in the hippocampus, basal forebrain and corpus callosum. Paraformaldehyde perfused rat brains were sectioned at 30 µm and stained with either IBA-1 antibody to label all microglia or Ox-6 antibody to label activated microglia in the hippocampus, basal forebrain and corpus callosum. (**A**) Photomicrographs of the hippocampus (**top two panels**) basal forebrain (**middle two panels**) and corpus callosum (**bottom two panels**) in in coronal rat brain sections from AβO-injected and PBS-injected (control) rats 21 days post-injection. Scale bars are 100 µm or 1 mm as indicated; (**B**) quantification using optical densitometry (IBA-1) or activated microglia cell counts (Ox-6) from three adjacent tissue sections per animal. No differences in IBA-1 labelling were observed between AβO-injected and controls across all brain regions. AβO-injected rats had significantly more Ox-6 positive cells within the corpus callosum compared to controls 21 days post-injection. Data presented as group means ± SEM. * indicates statistical significance between AβO-injected and control rats using 2-way ANOVA followed by Tukey’s post-hoc analysis, *p* < 0.05, *n* = 5 for each experimental group.

One of the earliest pathological consequences in the pathogenesis of AD includes an increased microglial response within several brain regions including the cortex, hippocampus, basal forebrain and white matter tracts [39]. To assess microglia activation an antibody specific to activated microglia (Ox-6) along with an antibody that labels all microglia (IBA-1) regardless of activation status was used (Figure 4). No significant differences were observed in either total microglia or activated microglia within the hippocampus or basal forebrain between AβO-injected and controls (Figure 4). However, a significant increase in Ox-6 positive activated microglia was observed in AβO-injected rats at 21 days post-injection within the corpus callosum compared to control rats (Figure 4, control—6.5 ± 1.8 cells/mm^2^, AβO—14.11 ± 3.40 cells/mm^2^).

## 3. Discussion

The early progression of AD still remains elusive. AβO are potent neurotoxic proteins that may play a key role in this process. In vivo models of disease can help the field gain a better understanding on the effects of AβO in disease pathogenesis. This study aimed to examine the effects of a single acute exposure of AβO in the rat. To achieve this, rats were injected bilaterally with AβO into the lateral ventricles and allowed to survive to multiple endpoints. Our results revealed a transient perturbation in learning and memory as well as mild anxiety-like behaviours. These behavioural deficits corresponded with a transient increase of Aβ within the corpus callosum and cingulate gyrus, an increase in activated microglia within these same areas and a decrease in cholinergic neurons within the basal forebrain.

The Morris water maze task is commonly used to test in learning and memory impairment in rodent models of AD [40,41,42]. In the present study, we demonstrate that an acute exposure to AβO induced transient behavioural learning and memory deficits. We showed that rats exposed to AβO were slower to learn the task initially (day 9) and but performed to the same level as controls by day 11. To test memory, probe trials were performed on days 12 and 19. AβO-injected rats only demonstrated impairment on day 12 and recovered on the test performance by day 19. Overall, these data suggest that the AβO injections caused a mild and transient impairment in learning and memory. Previous studies using acute single injection of Aβ peptides have shown similar acute deficits [43] indicating a potential role of these peptides in inducing transient impairments in memory and learning.

The open field task is used to analyze exploratory and anxiety-like behaviour [44]. Our results demonstrated that AβO-injected rats moved less and spent less time in the central zone. This significant difference may suggests anxiety-like behaviour [44]. More importantly, anxiety is an early clinical symptom commonly observed in patients diagnosed with AD. Similar anxiety-like behaviour was also observed in a transgenic rat carrying the Swedish human APP mutation (Tg6590). This rat also demonstrated increased levels of AβO within the hippocampus and cortex [45]. An additional study using this rat line also demonstrated both anxiety-like behaviour with the open field task, as well as deficits in spatial learning and memory with the Morris water maze [46]. Combined with our data, this suggests that AβO may play a role in inducing anxiety-like behaviour in the rat.

Pathological analysis on AβO-injected rats revealed a transient increase in Aβ labeling in the corpus callosum and cingulate gyrus that resolved by 21 days. This finding is congruent with the timing in the observed transient deficit in learning and memory. This indicates that the Aβ did indeed get into the brain parenchyma but was likely eventually cleared the brain parenchyma. It is possible, although unlikely given its anatomical location that the observed increase in Aβ was endogenous rather than from the injected peptides. In another study, using a chronic injection approach in which the group injected 1 µg of AβO 3 times per week for a total of 15 injections (total 15 µg of AβO compared to our 23 µg single injection paradigm), they found positive Aβ labelling within the hippocampus, entorhinal cortex, amygdala, and striatum [47]. These same regions were also identified in a study characterizing spatio-temporal progression of Aβ deposition in human AD brains [48] indicating that a chronic injection paradigm may recapitulate more aspects of the human disease than our single injection paradigm. Our rationale for injecting 23 µg was to have the ability to inject the most amount of AβO possible in a single bolus without using excessive injection volume. In our hands, a higher amount of AβO results in precipitation of the peptide out of solution. We chose to use a single injection paradigm to reduce the potential for multiple inflammatory injuries seen with either chronic injections or multiple infusions. Moreover, by using a single injection paradigm, we could test if a single exposure of AβO could seed further generation and deposition of AβO in the rat brain. Our results in this study indicate that this was not the case. Our study, however, may potentially add a critical piece of information currently lacking in the field. In both human AD patients and AD animal models, increased deposition of AβO within the brain parenchyma have correlated with decreased AβO levels in cerebrospinal fluid (CSF) [36,47,49,50,51]. These studies suggest a connection between the levels of AβO within the brain parenchyma and in the CSF. Further studies will need to carefully examine this relationship between brain parenchymal AβO and CSF AβO levels. Moreover, our study was the first to describe the resulting loss of cholinergic neurons within the basal forebrain. This is a key finding since a key pathological feature that defines AD cases in humans is cholinergic neuron loss within the basal forebrain [38]. Our in vivo findings need to be followed up with mechanistic studies aimed at understanding how AβO exposure directly targets cholinergic neurons.

Although activated microglial cells seem to play a role in the pathogenesis of the disease, the effects on the outcome of the disease is uncertain. Different studies have concluded contradictory roles for activated microglia, specifically, whether it exacerbates or attenuates the neurotoxic effects of AβO [52,53]. Results from our study demonstrated an increase in activated microglia within the corpus callosum of AβO injected rats. It was interesting that this difference was only observed 21 days following injury. It is possible that the deposition and clearance of AβO left an additional injury resulting in this delayed microglia activation. Future studies need to be done to better understand the role of microglia activation in response to AβO exposure in vivo. This will help shed light on the harmful vs. helpful role of microglia in AD.

In summary, our study found that exposure of AβO into the rat brain resulted in transient deficits in spatial learning memory with a concomitant observation of anxiety-like behaviour. These behavioural deficits were concomitant a transient increase in Aβ deposition which was eventually cleared. The single exposure to AβO also resulted in microglia activation in the corpus callosum indicating that AβO can elicit inflammation in the white matter tracts. Future studies need to further elucidate the mechanism responsible, specifically for the loss of cholinergic neurons in response to AβO exposure. By varying the dose and timing of exposure, it’s possible to gain further insight into these mechanisms. Overall, results from this study and others suggest that exposure to AβO in the rat may be a useful tool in assessing the early phases for the pathogenesis of AD.

## 4. Materials and Methods

### 4.1. Synthetic AβO Preparation

AβO were prepared as previously described [47]. Briefly, Aβ_1-42_ peptide (Bachem, Bubendorf, Switzerland) was purchased and stored at −20 °C until use. Ice-cold 1,1,1,3,3,3-hexafluro-2-propanol (HFIP, Sigma Aldrich, St. Louis, MO, USA) was added to dissolve the peptide and make a final of 1 mM. The solution was vortexed and aliquoted into microcentrifuge tubes with 10 µL each. Tubes were air-dried on ice for 15 min and then lyophilized for 1 h. Resulting peptide was stored at −80 °C until use. Prior to surgery, peptides were re-dissolved in 10 µL of anhydrous dimethyl sulfoxide (DMSO, BioShop, Burlington, ON, Canada), sonicated for 10 mins at 37 °C, and 10 mM phosphate buffered saline (PBS, pH 7.4) was added to make a final concentration of 150 µM. Immediately after adding PBS, peptide solution was incubated at 4 °C for 24 h.

### 4.2. Experimental Groups and Surgical Procedures

All animal protocols were approved by Western University Animal Care Committee. Six-month old male Wistar rats (Charles River, Montreal, QC, Canada) were used for this study. Each rat was housed individually with food and water provided ad libitum. Rats were randomly assigned to either an experimental or a control group. Rats were also allowed to habituate to their new environment for at least one week prior to surgeries.

Rats were anaesthetized with 3% isoflurane (Baxter Corporation, Mississauga, ON, Canada) and fixed onto a stereotaxic apparatus. Rats were kept under isoflurane throughout the surgery, body temperature was maintained at 37 °C using a heating pad. Bilateral intracerebroventricular (ICV, AP: −0.8 mm, ML: ±1.4 mm, DV: −4.0 mm) injections were performed using a Hamilton syringe (Hamilton, Reno, NV, USA). AβO or 10 mM phosphate buffered saline (PBS—control, pH 7.4) was injected into each ventricle (17 µL per ventricle—23 µg peptide total) at a rate µL/min. Rats were given buprenorphine (0.1 mL/100 g body weight—Reckitt Benckiser Healthcare, Oakville, ON, Canada) subcutaneously and 0.03 mL Baytril (Bayer, Toronto, ON, Canada) intramuscularly. Following surgery, rats were monitored in a cage under a heat lamp until recovery from anaesthesia. A total of eight rat groups were used in this study. Rats were injected with either AβO or PBS and were euthanized at 1 (*n* = 5), 3 (*n* = 5), 7 (*n* = 5), or 21 (*n* = 8) days post-surgery. Rats euthanized at 21-days post-surgery underwent behavioural studies as described below. No differences in body weight changes were observed between the surgery and post-surgery day 21 (control—+1.2 ± 1.4 g, AβO—+0.8 ± 1.1 g).

### 4.3. Behavioural Assessments

Morris water maze was performed to determine performance in spatial learning and memory. After surgery, rats were allowed to recover for one week before behavioural assessments began. Rats were subjected to four days of spatial learning between post-surgery days 8–11 and received four trials per day. During spatial learning, rats were given 90 s to find the submerged platform for each trial. If unsuccessful within the allotted time, rats would be guided to the platform. After each trial rats would remain on the platform for 30 s. To begin each trial, the rat placement within the pool was randomly selected. Rats were subjected to one probe trial per day on post surgery day 12 and 19 to test for short and long-term spatial memory respectively. In these trials, rats were allowed to freely swim around the pool for 30 s while the platform was taken out. The rats were subjected to four trials per day to find the platform. The purpose of the cued learning trial was to control for the rats’ ability to recognize a visual cue and their ability to swim towards the platform. All experiments were tracked using video-tracking software (ANY-maze, Wood Dale, IL, USA). Latency to reach target zone, latency to reach platform zone and mean swim speeds were measured.

Open field analysis (OF, Med Associates Inc., Fairfax, VA, USA) was used to assess exploratory and anxiety-like behaviours. Immediately after their last cued learning trial on post-surgery day 21, rats were moved to the room where OF was performed. Rats were allowed to rest and acclimate to the environment for at least 4 h prior to OF analysis. Boxes were cleaned with ethanol before and after each use. One rat was placed at the front and center of each box and allowed to explore for 20 min. Ambulatory time, ambulatory distance, and vertical counts were measured.

### 4.4. Pathological Analysis

Rats were euthanized by injecting a lethal dose of Euthanyl (Bimeda-MTC, Cambridge, ON, Canada) intraperitoneally. Rats were perfused trans-aortically with 10 mM PBS for 3 min followed by 4% paraformaldehyde for 7 min. Brains were removed and placed in 4% PFA and stored at 4 °C for 24 h. Brains were then transferred to 30% sucrose at 4 °C for 72 h. Thirty µm coronal brain sections were sliced from the rat brain tissue using a CryoStar Nx50 (Thermo Scientific, Waltham, MA, USA). Sections were stored in cryoprotectant (30% sucrose, phosphate buffer pH = 7.2, and ethylene glycol) at −20 °C until use. For immunohistochemistry labeling of Aβ, Ox6 and ChAT, brain sections were washed in 10 mM PBS to remove cryoprotectant. Endogenous peroxidase activity was then blocked by incubating sections with 1% hydrogen peroxide (Fisher Chemicals, Fair Lawn, NJ, USA). Sections were then washed 3 × 5 min with PBS and incubated at room temperature for 1 h in secondary antibody species raised specific serum (1:500) diluted in 10 mM PBS. Sections were then washed in 10 mM PBS (3 × 5 min) followed by incubation overnight at 4 °C with either anti-mouse Aβ4G8 (1:500, Covance, Princeton, NJ, USA) which detects the 17–24 AA sequence of the Aβ peptide, anti-mouse Ox-6 (1:1000, Pharmingen, San Jose, CA, USA) which detects the major histocompatibility complex (MHC) class II of activated microglia, anti-rabbit IBA-1 (1:1000, Wako, Richmond, VA, USA) which detects the ionized calcium binding adapter molecule of all microglia or anti-mouse choline acetyltransferase (ChAT, 1:500, Abcam, Cambridge, MA, USA) which detects cholinergic neurons via the anti-choline acetyltransferase enzyme. Sections were incubated at their respective concentrations diluted in 10 mM PBS with 0.2% Triton X-100. Sections were then washed in 10 mM PBS (3 × 5 min) followed by incubation in horse radish peroxidase tagged donkey anti-mouse or donkey anti-rabbit secondary antibodies diluted in serum and 10 mM PBS for 1 h at room temperature. Sections were then washed in 10 mM PBS (3 × 5 min) followed by incubation in 2% avidin-biotin complex (Vectastain Elite ABC Kit, Vector Laboratories, Inc., Burlingame, CA, USA) for one hour at room temperature. Finally, sections were incubated in 0.05% 3,3′-diaminobenzidine tetrahydrochloride (DAB; Sigma, Toronto, ON, Canada) and 1% H_2_O_2_ for 15 min at room temperature. Sections were dehydrated in increasing concentrations of alcohol, cleared in xylene, and mounted with Depex mounting medium (Depex, BDH Chemicals, Poole, UK). To ensure consistency, sections from different surgical groups were processed at the same time.

### 4.5. Imaging Investigators Were Blinded to Surgical Identity for All Image Acquisition

Imaging was performed using a Nikon Eclipse Ni-U upright microscope with a DS-Fi2 high definition color camera and imaging software NIS Elements Colour Camera (Nikon Instruments, Melville, NY, USA). For all optimal densitometry analysis, images were taken at the same exposure time. Optical densitometry was performed on a minimum of three 8-bit scale grey images per region per animal (ImageJ64 software, National Institute of Health, Bethesda, MD, USA).

### 4.6. Statistical Analysis

Investigators were blinded to surgical group identities for all analysis. Statistical analysis was completed using GraphPad Prism 6.0 (La Jolla, CA, USA). Either a student’s *t* tests, one-way or two-way analysis of variance (ANOVA) followed by Tukey’s multiple comparisons post-hoc tests were performed where appropriate. Data are expressed as means ± standard error of the mean (SEM). An asterisk (*) indicates statistical significance between group means with *p* < 0.05.
